# Insulin receptor expression and its association with hyperinsulinemia in triple negative breast cancer

**DOI:** 10.1210/jendso/bvag041

**Published:** 2026-02-17

**Authors:** Alexis J Engel, Krupa Samuel, Ilana R Bass, Sylvia Lin, Irini Markella Antoniou, Radhi Yagnik, Elisa Port, Sheldon M Feldman, Neil B Friedman, Susan K Boolbol, Brigid Killelea, Melissa Pilewskie, Lydia Choi, Christopher A Galifi, Teresa L Wood, Nathan G Kase, Derek LeRoith, Nina A Bickell, Emily J Gallagher

**Affiliations:** Department of Medical Education, Icahn School of Medicine at Mount Sinai, New York, NY 10029, USA; Department of Surgery, Icahn School of Medicine at Mount Sinai, New York, NY 10029, USA; Division of Endocrinology, Diabetes and Bone Diseases, Department of Medicine, Icahn School of Medicine at Mount Sinai, New York, NY 10029, USA; Department of Population Health Science and Policy, Center for Health Equity and Community Engaged Research, Icahn School of Medicine at Mount Sinai, New York, NY 10029, USA; Division of Endocrinology, Diabetes and Bone Diseases, Department of Medicine, Icahn School of Medicine at Mount Sinai, New York, NY 10029, USA; Department of Population Health Science and Policy, Center for Health Equity and Community Engaged Research, Icahn School of Medicine at Mount Sinai, New York, NY 10029, USA; Department of Surgery, Icahn School of Medicine at Mount Sinai, New York, NY 10029, USA; Department of Surgery, Montefiore Medical Center, Albert Einstein College of Medicine, New York, NY 10461, USA; Department of Surgery, Mercy Medical Center, Baltimore, MD 21202, USA; Nuvance Health, Poughkeepsie, NY 12601, USA; Department of Surgery, Brigham and Women's Hospital, Boston, MA 02115, USA; Department of Surgery, Memorial Sloan Kettering Cancer Center, New York, NY 10065, USA; Department of Surgery, Wayne State University School of Medicine, Detroit, MI 48201, USA; Department of Pharmacology, Physiology and Neuroscience, Center for Cell Signaling and Cancer Institute of New Jersey, Rutgers Biomedical and Health Sciences, Newark, NJ 07039, USA; Department of Pharmacology, Physiology and Neuroscience, Center for Cell Signaling and Cancer Institute of New Jersey, Rutgers Biomedical and Health Sciences, Newark, NJ 07039, USA; Department of Obstetrics, Gynecology, and Reproductive Science, Icahn School of Medicine at Mount Sinai, New York, NY 10029, USA; Division of Endocrinology, Diabetes and Bone Diseases, Department of Medicine, Icahn School of Medicine at Mount Sinai, New York, NY 10029, USA; Tisch Cancer Institute at Mount Sinai, Icahn School of Medicine at Mount Sinai, New York, NY 10029, USA; Department of Population Health Science and Policy, Center for Health Equity and Community Engaged Research, Icahn School of Medicine at Mount Sinai, New York, NY 10029, USA; Tisch Cancer Institute at Mount Sinai, Icahn School of Medicine at Mount Sinai, New York, NY 10029, USA; Institute for Health Equity Research, Icahn School of Medicine at Mount Sinai, New York, NY 10029, USA; Division of Endocrinology, Diabetes and Bone Diseases, Department of Medicine, Icahn School of Medicine at Mount Sinai, New York, NY 10029, USA; Tisch Cancer Institute at Mount Sinai, Icahn School of Medicine at Mount Sinai, New York, NY 10029, USA

**Keywords:** insulin receptor, hyperinsulinemia, triple negative breast cancer

## Abstract

**Purpose:**

Hyperinsulinemia and tumor insulin receptor (IR) expression have been associated with triple negative breast cancer (TNBC) progression in preclinical models. We aimed to evaluate the expression of the IR, IGF-1 receptor (IGF-1R), and associated signaling protein expression in TNBC and their correlations with demographic and metabolic parameters in a population of women with TNBC.

**Methods:**

We identified cases of TNBC from our multi-institutional, cross-sectional study of self-identified Black and White women with newly diagnosed breast cancer. Survey, anthropometric, screening behavior, laboratory, and tumor pathology reports were collected, along with formalin-fixed paraffin embedded tumor samples. We performed immunohistochemistry (IHC) analysis and quantified the expression of IR, IGF-1R, phosphorylated Erk1/2 (pErk1/2), and FOXO3a. Clinical information was correlated with IHC scoring.

**Results:**

There were 93 TNBC cases. IHC staining and quantification found that 63% of TNBC cases stained positive for IR, 73% for IGF-1R, 67% for FOXO3a, and 43% for pErk1/2. Positive IR staining was more prevalent in Black women than White women (*P* = .003) and was associated with body mass index and fasting insulin on univariate analysis but was not significantly associated with age. On multivariate analysis, IR expression was associated with fasting insulin but not race. IGF-1R, FOXO3a, and pErk1/2 staining were not associated with any of these factors.

**Conclusion:**

Tumor IR expression was associated with higher fasting insulin, and higher fasting insulin was more prevalent among Black women. Further studies are needed to determine the importance of hyperinsulinemia and tumor IR expression in the development of TNBC.

In the United States, racial disparities exist in breast cancer incidence and survival. Despite a lower incidence of breast cancer among Black women than White women, Black women tend to be diagnosed at a younger age and have 81% higher rates of estrogen receptor (ER)-negative, progesterone receptor-negative, nonhuman epidermal growth factor receptor 2 (HER2) overexpressing breast cancer, also known as triple negative breast cancer (TNBC) [1-4]. TNBC is an aggressive breast cancer subtype that carries a worse prognosis than other breast cancer subtypes [1]. A large part of the disparity in overall breast cancer outcomes between Black women and White women relates to higher rates of TNBC among Black women and unequal access to screening, treatment, and medical care [5]. However, TNBC can be diagnosed between mammography screening tests and can present at a later stage than ER/progesterone receptor-positive tumors, therefore leading to worse outcomes [1, 6]. Among women diagnosed with TNBC, Black women have an approximately 16% to 28% greater breast cancer mortality in comparison to White women after adjustment for demographic, clinical, and treatment factors [7]. So, even accounting for the higher risk of developing TNBC, Black women still have worse outcomes than White women with TNBC. Therefore, there is a need to understand the factors contributing to the disparities related to biology in TNBC.

Obesity, type 2 diabetes, and metabolic syndrome have all been associated with a greater risk of developing TNBC, and these conditions are more prevalent in Black women in the United States than White women [8, 9]. Although obesity is frequently measured by body mass index (BMI) in epidemiology research, BMI is an imperfect measure of systemic metabolic health. Insulin resistance and hyperinsulinemia frequently co-exist with obesity, are hallmarks of metabolic syndrome, and predate the development of hyperglycemia with type 2 diabetes. Preclinical studies have previously found that hyperinsulinemia leads to increased growth and metastasis in models of TNBC [10, 11]. Hyperinsulinemia was found to increase the expression of the transcription factor myc and to alter histone acetylation in TNBC models, leading to upregulation of myc-target genes as well as genes involved in ribosomal biogenesis [10, 12]. Recent preclinical studies have also proposed that adipocyte-derived and plasma exosomes promote TNBC progression in hyperinsulinemic models [13, 14]. In a syngeneic TNBC model and a human xenograft model, RNA-guided silencing of the insulin receptor (IR) curtailed primary tumor growth and lung metastasis in both normo- and hyperinsulinemic mice, accompanied by reductions in Akt and Erk1/2 signaling, decreased myc expression, along with lower expression of a number of transcription factors related to epithelial-to-mesenchymal transition [15, 16]. Myc activation signatures in breast cancer have been associated with decreased survival [17]. Based on these preclinical studies, we hypothesize that hyperinsulinemia contributes to the development of TNBC in humans through its action on the IR.

We previously found that insulin resistance mediates part of the association between race and breast cancer prognosis across all breast cancer subtypes [18]. Performing immunohistochemistry (IHC) staining of hormone receptor-positive and -negative tumors revealed that breast cancers from Black women had higher IR expression and a higher IR/IGF-1 receptor (IGF-1R) ratio than breast cancers from White women. In that study, only 11 of the 196 tumor specimens we examined were from women with TNBC, and only 3 of the 11 TNBC specimens were from Black women [18]. In our current study, we evaluated the relationships between IR, IGF-1R expression, and signaling proteins in TNBC with race and metabolic parameters.

## Materials and methods

### Patient accrual, data collection, and laboratory measurements

In this multi-institutional, cross-sectional study, institutional review board approval was obtained from all participating sites (Icahn School of Medicine at Mount Sinai, NY; Columbia University Medical Center, NY; Memorial Sloan Kettering Cancer Center, NY; Mercy Medical Center, MD; Yale University, CT; Wayne State University, MI; Newark Beth Israel Medical Center, NJ). Informed consent was obtained from all study participants. Inclusion criteria included self-identified Black (including Hispanic Black) women or White (excluding Hispanic White) women aged 21 years or older with a new diagnosis of breast cancer who had not undergone surgery or neoadjuvant chemotherapy prior to enrollment. Exclusion criteria included type 1 or type 2 diabetes treated with oral or injectable medication, except longstanding metformin; previous bariatric surgery; end-stage renal disease; hepatic cirrhosis; organ transplant; glucocorticoid treatment within 2 weeks prior to blood or tissue sampling; and receipt of neoadjuvant chemotherapy or hormonal therapy prior to blood tests or tissue sampling [18, 19]. Self-reported clinical data as well as comorbidities were collected via interviewer-administered surveys. Anthropometric measurements and fasting blood samples were obtained at a study visit or prior to surgery. For individuals who received neoadjuvant chemotherapy, blood samples and anthropometric measurements were obtained prior to starting treatment, and core biopsy samples taken before starting neoadjuvant chemotherapy were used for IHC staining, rather than surgical samples.

The entire cohort included 1206 self-identified Black (n = 295) and White (n = 911) women. The study protocol, interim report, and analysis of the impact of hyperinsulinemia on mediating the association between race and breast cancer prognosis for the entire cohort were previously published [18-20]. Of that cohort of 1206 women, 109 women had TNBC (9% of total cohort): 53 Black women (18% of all Black women with breast cancer) and 56 White women (6% of all White women with breast cancer). These 109 women with TNBC are the subjects of this study.

Each patient had height and weight measurements obtained at the study visit, from which BMI was calculated. Waist circumference was measured according to the NHANES procedure [21]. Blood pressure was measured with a clinical electronic blood pressure monitor. Venous blood was drawn after an overnight fast (minimum 8 hours) to evaluate plasma glucose, serum insulin, C-peptide, and lipids (total cholesterol, high-density lipoprotein cholesterol, low-density lipoprotein cholesterol, and triglycerides). Insulin resistance was measured with the Homeostatic Model Assessment of Insulin Resistance (HOMA-IR) equation: [fasting glucose (mg/dL)×fasting serum insulin (μU/mL)]/405.

Clinical data included self-reported breast cancer screening, medical comorbidities, smoking, alcohol use, diet quality (poor or fair, good, very good, excellent), physical activity, education, income, health insurance, and family history of breast cancer. Breast cancer screening was considered inadequate if women aged 50 to 74 had no mammogram within 2 years prior to the mammogram leading to their current breast cancer diagnosis. The Charlson Comorbidity Index was calculated [22].

### Definitions of obesity, metabolic syndrome, and insulin resistance

Obesity was defined as a BMI of ≥30 kg/m^2^ or by the Adult Treatment Panel III waist circumference (WC) cutoff of ≥88 cm. The metabolic syndrome was defined as having 3 or more of the following 5 criteria: (1) WC ≥ 88 cm; (2) triglycerides ≥ 150 mg/dL or on treatment for hypertriglyceridemia; (3) high-density lipoprotein < 50 mg/dL; (4) fasting glucose ≥100 mg/dL; (5) systolic blood pressure ≥ 130 mmHg or diastolic blood pressure ≥ 85 mmHg or on treatment for hypertension [23]. Insulin resistance was defined as a HOMA-IR score of >2.8 [24].

### Breast cancer subtype and stage determination

Clinical pathology reports were accessed through the parent study protocol, which involved direct communication with the treating oncologists and pathology departments to obtain copies of the pathology reports that were used to identify cases of TNBC. As recommended by the American Joint Committee on Cancer, tumor grade was defined by the Nottingham grading system and stage according to the Eighth Edition American Joint Committee on Cancer Staging Manual [25].

### Immunohistochemistry staining and analysis

Of the 109 cases of TNBC, de-identified formalin-fixed paraffin embedded 5 um slides were cut by the pathology teams from 45 Black women and 48 White women (n = 93). Sixteen cases had very small tumors with insufficient tissue available for research purposes. Slides were stored at 4 °C until staining. The tissues were de-paraffinized by incubating in a 60 °C oven followed by xylenes washes and rehydrated in decreasing concentrations of ethanol, as previously described [18]. Heat- and chemical-induced antigen retrieval was performed by heating in a microwave in sodium citrate buffer (pH 6) for IR, FOXO3a, phosphorylated Erk1/2 (pErk1/2) (Thr202/Tyr204), and in tris-EDTA buffer (pH 9) for IGF-1R staining. After a 5-minute incubation with 3% hydrogen peroxide, blocking was performed for an hour at room temperature using 5% goat serum in Tris-buffered saline with 0.1% Tween 20 for the Cell Signaling antibodies or Abcam Protein Block for 10 minutes for the Abcam antibody. Cell Signaling antibodies were diluted in SignalStain diluent; the Abcam antibody was diluted in 10X phosphate-buffered saline and incubated overnight. Primary antibodies were anti-IR Ab (ab137747, Abcam, Cambridge, MA, RRID:AB_3717506) 1:300 dilution, anti-IGF-1R Ab (#3027, Cell Signaling, Danvers, CA, RRID:AB_2122378) 1:200 dilution, anti-FOXO3a Ab (#12829, Cell Signaling, RRID:AB_2636990) 1:3200 dilution, and anti-pErk1/2 (Thr202/Tyr204) Ab (#4370, Cell Signaling, RRID:AB_2315112) 1:400 dilution. SignalStain Boost IHC Detection reagent (Cell Signaling) or horseradish peroxidase conjugate (Abcam) incubation, followed by 3,3′-diaminobenzidine chromogen incubation were used for signal detection (Immpact DAB peroxidase substrate, VectorLabs, Burlingame, CA). Harris hematoxylin was used for nuclear counterstaining. The tissues were dehydrated in ethanol and xylene, covered with a coverslip and mounted with EUKITT mounting media (Electron Microscopy Sciences, Hatfield, PA).

Investigators independently quantified the expression of IR (n = 92), IGF-1R (n = 92), pErk1/2 (n = 91), and FOXO3a (n = 90) for intensity (none = 0, weak = 1, moderate = 2, intense = 3) and for localization of staining (nuclear vs nonnuclear). In subsequent analysis, 0 and 1 intensity IHC staining were considered negative, and 2 and 3 were considered positive. Initial quantification was performed independently by each reviewer (A.J.E., E.J.G.). Discrepancies were then assessed in a subsequent quantification analysis performed jointly by reviewers. Reviewers were blinded to clinical information.

### Statistical analysis

Patient characteristics and IHC analysis were described using basic statistics. Group comparisons were made using *t*-tests for continuous variables that followed a normal distribution, determined using the Shapiro-Wilk test, and were reported using means and SD. The Wilcoxon rank sum test was used for continuous variables that did not follow a normal distribution, and data were reported using medians and interquartile ranges (IQRs). Chi-squared or Fisher’s exact tests were used on categorical variables and reported as frequencies and proportions. Missing clinical data ranged from 2.2% for tumor size and stage to 25.8% for income. Missing data for any variable were excluded from the analysis of that specific variable. For multivariate logistic regression models, we employed a complete case analysis approach, in which observations with missing values for any covariate included in a given model were excluded from that model [26]. R software 4.4.1 and SPSS 29.0.2.0 were used for statistical analyses. Statistical significance was set to *P*-value < .05.

## Results

### Metabolic and tumor characteristics of women with TNBC

The characteristics of the self-identified Black and White women with TNBC are shown in Table 1. There was no statistically significant difference in age between the groups, but Black women had more than double the prevalence of obesity as defined by BMI (56%) compared with White women (20%, *P* = .001). The rates of abdominal obesity, defined by WC, were higher than the rates of obesity as defined by BMI in both groups of women. There was no difference in prevalence of family history of breast cancer between Black and White women.

**Table 1.** Characteristics of women with TNBC tumor tissue by self-identified race

	Total	White	Black	*P-*value
**n (%)**	93 (100)	48 (52)	45 (48)	
Age (years)	56.9 (13.6)	55.5 (14.9)	58.4 (12.1)	.281
**Metabolic characteristics**				
BMI (kg/m^2^)	27.6 [24.9-32.7]	26.2 [22.6-28.6]	30.6 [27.0-34.9]	**<**.**0001**
Obese (BMI ≥ 30 kg/m^2^) (%)	34/90 (37)	9/45 (20)	25/45 (56)	.**001**
Abdominal obesity (WC > 88 cm) (%)	59/90 (72)	25/45 (56)	37/45 (87)	.**001**
Metabolic syndrome (%)	23/87 (26)	8/43 (19)	15/44 (34)	.1
**Laboratory parameters**				
HbA1c (%)	5.7 [5.4-6.0]	5.5 [5.2-5.8]	5.8 [5.6-6.2]	.**001**
Cholesterol, total (mg/dL)	198 (37.9)	195 (36.2)	200 (39.9)	.983
HDL (mg/dL)	60 [50-69]	60 [51-78]	60 [50-67]	.272
LDL (mg/dL)	117 (35.2)	112 (33.2)	122 (37.1)	.239
Triglycerides (mg/dL)	85 [66-112]	90 [70-124]	88 [64-109]	.423
C-peptide (ng/mL)	1.9 [1.3-2.8]	1.9 [1.3-2.5]	1.9 [1.4-2.8]	.555
Insulin (µIU/mL)	6.3 [3.8-10.4]	5.2 [3.3-8.2]	8.1 [4.9-10.9]	.**028**
Glucose (mg/dL)	92 [86-101]	90 [88-99]	93 [86-106]	.349
IGFBP-1 (ng/mL)	8.9 [4.1-15.0]	11 [6.1-19.0]	6.3 [3.6-13.3]	.**003**
HOMA-IR	1.6 [0.8-2.4]	1.4 [0.7-2.0]	2.0 [1.1-2.9]	.**028**
**Tumor characteristics**				
Tumor size (cm)	1.4 [1.0-2.2]	1.3 [1.0-1.8]	1.6 [1.0-2.5]	.2
Stage (%)				.**022**
IB	54 (58)	34 (71)	20 (45)	
IIB	23 (25)	12 (25)	11 (24)	
IIIB	5 (3)	1 (2)	4 (9)	
IIIC	5 (5)	1 (2)	4 (9)	
Missing	6 (6)	0 (0)	6 (13)	
Grade (%)				
2	9 (10)	3 (6)	6 (13)	.248
3	84 (90)	45 (94)	39 (87)	
**Comorbidities**				
Charlson Comorbidity Index (≥1) (%)	60/92 (65)	23/47 (49)	37/45 (82)	.**001**
**Lifestyle/behavioral factors** (%)				
Mammogram ≥ 2 years before diagnosis	20/86 (23)	11/44 (25)	9/42 (21)	.822
Smoking: never smoker	57/87 (66)	24/44 (55)	33/43 (77)	.051
Alcohol: >2 drinks/week	18/83 (22)	15/44 (34)	3/39 (8)	.**010**
Diet: very good/excellent diet	35/87 (40)	26/45 (58)	9/42 (21)	.**001**
Family history of breast cancer	21/84 (25)	11/43 (26)	10/41 (24)	.85
**Socioeconomic factors** (%)				
Education: < college education	18/87 (21)	5/44 (11)	13/43 (30)	.056
Income: < $75 000/year	33/69 (41)	9/37 (24)	24/32 (59)	**<**.**001**
Insurance: commercial insurance	67/86 (78)	38/44 (86)	29/42 (69)	.09

Percentages and statistical testing were performed for each variable using the available denominator. Data are presented as number and percent, mean and SD, or median and quartile 1 to quartile 3 as indicated. Bold font in the *P*-values indicates values that were considered statistically significant.

Abbreviations: BMI, body mass index; HbA1c, hemoglobin A1c; HDL, high-density lipoprotein; HOMA-IR, Homeostatic Model Assessment of Insulin Resistance; IGFBP1, IGF binding protein 1; LDL, low-density lipoprotein; TNBC, triple negative breast cancer; WC, waist circumference.

Despite the higher prevalence of obesity in Black women, there were no statistically significant differences in lipid profiles, fasting glucose, or C-peptide levels. However, median hemoglobin A1c, which reflects longer-term glycemic control, was higher in Black women (5.8 [IQR: 0.6]%) than in White women (5.5 [IQR: 0.6]%, *P* = .001). Black women had higher levels of fasting insulin compared with White women (8.1 [IQR: 6.5] µIU/mL vs 4.9 [IQR: 5.0] µIU/mL, *P* = .035), along with HOMA-IR (2.0 [IQR: 2.0] vs 1.3 [IQR: 1.2], *P* = .046) and lower levels of IGF binding protein 1 (6.3 [IQR: 10.3] ng/mL vs 11 [IQR:12.9] ng/mL, *P* = .004). Black women also had higher scores on the Charlson Comorbidity Index. With regard to social determinants of health, we found statistically significant differences in income, alcohol intake, and self-reported diet between Black women and White women with TNBC. There was no significant difference in primary tumor size or tumor grade. While there was no difference in screening behaviors, White women had more earlier-stage TNBC at diagnosis.

### Correlations between IHC expression and metabolic and tumor characteristics

**Figure 1.**
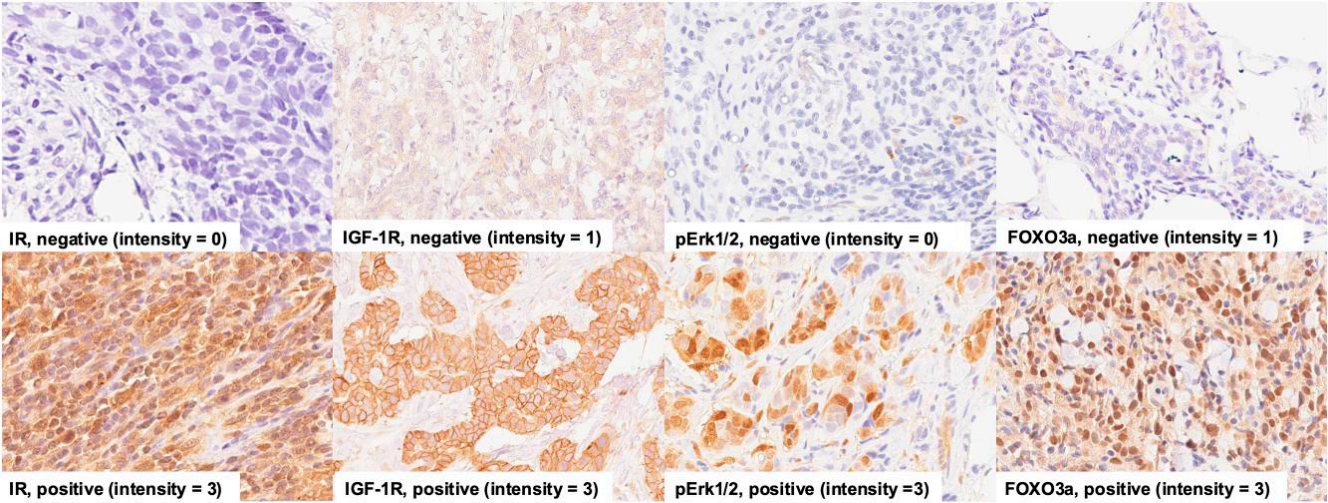
Representative images of IHC staining. IHC staining of TNBC specimens was performed as described in the Methods section with antibodies to IR, IGF-1R, pErk1/2, and FOXO3a. The images are shown in 40× magnification. Abbreviations: IGF-1R, IGR-1 receptor; IHC, immunohistochemistry; IR, insulin receptor; pErk1/2, phosphorylated Erk1/2; TNBC, triple negative breast cancer.

IR, IGF-1R, FOXO3a, and pErk1/2 IHC staining was performed on tumor samples from 93 women with TNBC. The range of staining for each antibody is shown in Fig. S1 [27]. Forty-five of these tumor specimens were from Black women (48%) and 48 were from White women (52%). A total of 90 tissues were stained and analyzed for IR, IGF-1R, and pErk1/2 and 89 for FOXO3a expression. Each total is less than 93 because some specimens had inadequate tumor tissue on the slides to allow for analysis. Eighty-eight tissues (95%) stained positive for 1 or more markers. Positive staining for IR was seen in 63% (n = 57/90), IGF-1R in 73% (n = 66/90), FOXO3a in 67% (n = 60/89), and pErk1/2 in 43% (n = 39/90). Representative images of IR staining are shown in Fig. 1.

In univariate analysis, positive IR staining was more prevalent in Black women than White women (*P* = .005) but was not significantly associated with age. Positive IR staining was associated with higher BMI (*P* = .011), fasting insulin (*P* = .003), C-peptide (*P* = .028), and HOMA-IR (*P* = .005) in univariate analysis (Table 2). HOMA-IR, C-peptide, and fasting insulin were highly correlated. Therefore, fasting insulin was used in the binary logistic regression model. When fasting insulin and race were included in the model, insulin was significantly associated with positive IR expression [hazard ratio (HR) 1.15; 95% confidence interval (CI) 1.007-1.307] but not race (HR 2.39; 95% CI 0.830-6.894). When BMI was also added to the model, insulin was no longer significantly associated with IR expression (HR: 1.133; 95% CI 0.984-1.305), and BMI was not significantly associated with IR expression (HR 1.026; 95% CI 0.913-1.152). Positive IGF-1R, FOXO3a, and pErk1/2 staining were not associated with any of these factors (Table S1A-1C [27]). We found that the ratio of IR/IGF-1R was positively associated with higher fasting insulin (*P* = .017) and HOMA-IR (*P* = .017) but was not significantly associated with race, age, BMI, or WC in univariate analysis (Table 3).

**Table 2.** Patient and tumor characteristics based on IR IHC expression

	Positive IR (n = 57)	Negative IR (n = 33)	*P*-value
Race, n (%)			.**005**
Black	33 (78.6)	9 (21.4)	
White	24 (50.0)	24 (50.0)	
Age (years)	57.8 (13.4)	56.8 (14.6)	.738
BMI (kg/m^2^)	28.5 [25.9-33.5]	26.9 [22.6-30.4]	.**011**
Waist circumference (cm)	101 [90.1-110.5]	92 [83.9-103]	.084
Fasting insulin (μIU/mL)	6.9 [4.9-10.6]	3.9 [2.6-6.9]	.**003**
HOMA-IR	1.8 [1.3-2.7]	0.9 [0.5-1.6]	.**005**
C**-**peptide (ng/mL)	2.0 [1.5-2.9]	1.5 [1.0-2.2]	.**028**
Metabolic syndrome, n (%)	14/52 (26.9)	7/31 (22.6)	.80

Data are presented as number and percent of row, mean and SD, or median and quartile 1 to quartile 3. Bold font in the *P*-values indicates values that were considered statistically significant.

Abbreviations: BMI, body mass index; HOMA-IR, Homeostatic Model Assessment of Insulin Resistance; IHC, immunohistochemistry; IR, insulin receptor.

**Table 3.** Patient and tumor characteristics based on IR/IGF-1R IHC expression ratio

	IR/IGF <1 (n = 21)	IR/IGF > 1 (n = 11)	*P*-value	IR/IGF =1 (n = 57)
Race, n (%)			.07	
Black	6 (28.6)	7 (63.6)		28 (49.1)
White	15 (71.4)	4 (36.4)		29 (50.9)
Age (years)	55.1 (13.7)	60.9	.28	57.5 (13.8)
BMI (kg/m^2^)	27.7 [22.3-31.2]	28.4 [25.3-33.9]	.44	27.5 [25.0-31.9]
Waist circumference (cm)	92 [89-103]	100 [95.8-105.5]	.31	99 [87.8-110]
Fasting insulin (μIU/mL)	4.2 [2.6-7.9]	9.3 [5.3-13.4]	.**017**	6.3 [3.6-9.8]
HOMA-IR	1.0 [0.6-2.0]	2.1 [1.6-3.3]	.**017**	1.4 [0.7-2.4]
Metabolic syndrome, n (%)	7/21 (33.3)	5/11 (45.5)	.61	10/51 (19.6)

Data are presented as number and percent, mean and SD, or median and quartile 1 to quartile 3. Samples with equal IR/IGF-1R IHC expression scores are excluded from the analysis. Bold font in the *P*-values indicates values that were considered statistically significant.

Abbreviations: BMI, body mass index; HOMA-IR, Homeostatic Model Assessment of Insulin Resistance; IGF-1R, IGF-1 receptor; IHC, immunohistochemistry; IR, insulin receptor.

### Nuclear IR expression in TNBC

**Figure 2.**
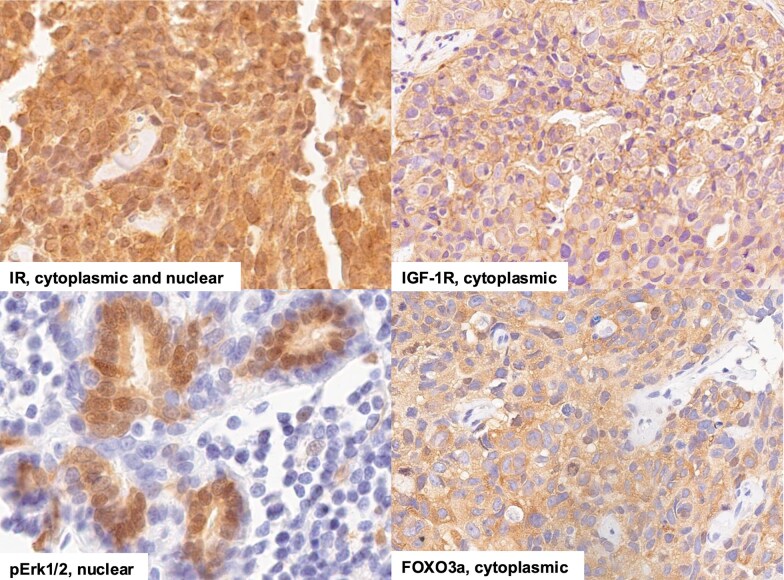
Representative images of protein localization for IR, IGF1R, FOXO3a, and pErk1/2. IHC staining of TNBC specimens was performed and evaluated for localization of proteins. The images are shown in 40× magnification. Abbreviations: IGF-1R, IGR-1 receptor; IHC, immunohistochemistry; IR, insulin receptor; pErk1/2, phosphorylated Erk1/2; TNBC, triple negative breast cancer.

We noted that of the samples that stained positive (2+ or 3+) for IR, the staining localized to the nucleus in 32% (n = 19/59). Nuclear IR expression was not associated with metabolic parameters in this group; however, if those with weak (1+) staining were also included, nuclear IR was associated with the prevalence of metabolic syndrome. Evaluating nuclear IR staining as a continuous variable, the intensity of nuclear IR staining was greater in Black women than White women (*P* = .003). For IGF-1R, 3.0% (n = 2/66) had positive nuclear staining. For FOXO3a, nuclear staining was found in 77% (n = 46/60) of samples. For samples that were positive for pErk1/2, 90% (n = 35/39) had positive nuclear staining (Table S2 [27]). The localization of IGF-1R, pErk1/2 and FOXO3a to the nucleus was not associated with race, age, or any metabolic factors. Representative images of nuclear and cytoplasmic staining are shown in Fig. 2.

## Discussion

In this study, we found that self-identified Black women with newly diagnosed TNBC had higher BMI, WC, hemoglobin A1c, fasting insulin, and HOMA-IR and more comorbidities than White women with TNBC. Similar screening behaviors were found in Black and White women, but earlier-stage cancer was found in White women. Positive tumor IR staining was more prevalent in Black women than White women, but in multivariate analysis, positive IR staining was significantly associated with fasting insulin but not race. Similarly, the tumor IR/IGF-1R ratio was associated with fasting insulin but not other factors.

Our findings are consistent with previous studies that have reported that Black women with newly diagnosed breast cancer are more likely to have conditions associated with hyperinsulinemia, such as metabolic syndrome [28, 29]. Interestingly, we found elevated fasting insulin in Black women compared with White women with TNBC, but there was no difference in fasting C-peptide levels between the 2 groups. This is consistent with previous research showing reduced hepatic but not extrahepatic insulin clearance in African American compared with European American women, contributing to systemic hyperinsulinemia [30]. Upon analyzing the variables associated with tumor IR expression, we found in the multivariable analysis that fasting insulin levels remained statistically associated with IR, independent of race. Additionally, we found that the IR/IGF-1R ratio was associated with fasting insulin. Prior studies have found that TNBC has downregulated IGF-1R expression compared with ER-positive breast cancers, although we found positive IGF-1R staining in almost three-quarters of our cases [31]. Studies have also found that the ratio of IR/IGF-1R can predict the sensitivity of cancer cells to insulin [32]. Therefore, it is possible that this association between fasting insulin and tumor IR expression indicates that TNBC may be more likely to develop in individuals with higher fasting insulin, where the cells express the IR. This remains to be proven, and it is as yet unknown if hyperinsulinemia causes an increase in IR expression or the IR/IGF-1R ratio. We found no association between race, metabolic factors, and expression or localization of pErk1/2 or FOXO3a staining, suggesting that these signaling proteins are not critical to hyperinsulinemia-associated TNBC development or progression.

**Figure 3.**
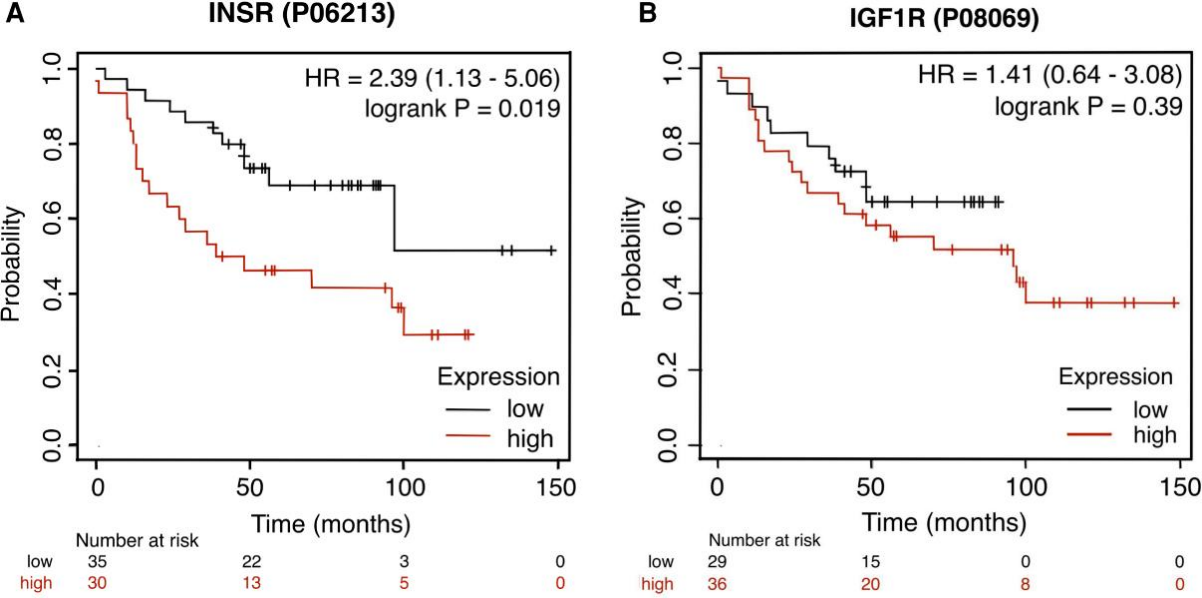
Overall survival for women with breast cancer based on IR and IGF-1R protein expression in KMPlot.com. KMPlot.com was used to generate overall survival Kaplan-Meier plots. The dataset used was not from our cohort but from Tang and colleagues [17]; n = 65 patients had IR (A) and IGF1R (B) protein expression available. The patients were split using the “auto select best cutoff” percentile option in KMPlot. No other restrictions were added to the analysis. The plots show hazard ratios with 95% confidence intervals in parentheses [17]. Abbreviations: IGF-1R, IGR-1 receptor; IR, insulin receptor.

Recent preclinical studies have reported the importance of IR for the development of HER2-positive and hormone receptor-positive breast cancer but not their progression. However, those murine studies did not examine TNBC [33], and the results contrasted with prior human studies where high IR expression in breast cancer correlated with worse breast cancer outcomes [34]. Indeed, using KMPlot.com to evaluate the association between IR and IGF-1R protein expression and overall survival in breast cancer [17, 35], we found that IR was associated with significantly decreased overall survival (HR 2.39; 95% CI 1.13-5.06, *P* = .019), but IGF-1R expression was not (HR 1.41; 95% CI 0.64-3.08, *P* = .39) (Fig. 3). It is important to note that the tumors used for this analysis were not from our cohort and were not all TNBC but were from protein analysis performed by liquid chromatography-mass spectrometry rather than IHC, as described by Tang et al [17]. In another preclinical model, silencing the IR in a syngeneic model of TNBC, there was a reduction in tumor growth and metastasis, although these were injected tumor cells derived from a c-myc/vegf-a overexpressing model [15]. Human TNBC is known to be a heterogeneous disease, and therefore it is possible that higher IR expression may be associated with the development and progression of certain subtypes of TNBC, but this remains to be determined.

The IR exists as 2 isoforms that differ by 1 exon: exon 11, which is 12 amino acids in length. Exon 11 is spliced out in the IR A isoform (IR-A) and is retained in the IR B isoform (IR-B) [36]. IR-A is commonly associated with cancer cell proliferation, while the IR-B isoform is associated with the metabolic actions of insulin. There are currently no antibodies that can distinguish the IR-A and IR-B isoforms by IHC. Therefore, we could not determine the IR isoform expression in our study. There are now novel antisense oligonucleotides that target IR-A but not IR-B, which should enable us to develop a greater understanding of the role of IR-A in human physiology and disease [37].

The classical functions of the IR and IGF-1R are as receptor tyrosine kinases that lead to activation of the PI3K/Akt/FOXO and Ras/Raf/MAPK pathways, mediating metabolic and mitogenic responses [38]. While IR and IGF-1R signaling can activate the Akt and MAPK signaling pathways, we did not see an association between hyperinsulinemia or receptor expression and either FOXO3a or pErk1/2 expression. It is important to note that these pathways can also be activated independent of growth factor receptor signaling in TNBC by gain of function mutations in PI3K/Akt/FOXO or Ras/Raf/MAPK signaling pathways [39].

In contrast, less is understood about the noncanonical role of the IR and IGF-1R in the nucleus. We observed nuclear IR localization in almost a third of breast cancers with positive IR staining. Similarly, a study from Sweden reported nuclear IR in ∼24% of breast cancers [40]. The authors reported that nuclear IR was associated with younger age and lower BMI [40]. However, their ability to examine nuclear IR in TNBC was limited by missing HER2 data [40]. Nuclear IR was identified by insulin binding studies almost 50 years ago, yet its functional significance has remained elusive [41, 42]. In metabolic tissues, nuclear IR was also found to associate with RNA polymerase II, and promoter binding by IR was mediated by host cell factor −1, leading to the regulation of genes involved in lipid metabolism and protein synthesis [43]. Much remains to be discovered about the significance of nuclear IR in breast cancer development and progression in the presence and absence of systemic hyperinsulinemia [44].

This study has several limitations. As a result of the cross-sectional design, we were unable to assess the duration of insulin resistance prior to breast cancer diagnosis or any associations with long-term clinical outcomes. We were additionally unable to determine what causes higher IR expression in certain TNBC, if it is secondary to hyperinsulinemia, and if hyperinsulinemia is also associated with higher IR expression in normal breast tissue. The percentage of our cohort with obesity is less than that of the typical US population. One explanation for this is that we excluded all individuals with diabetes taking medications other than metformin. Furthermore, many study participants were recruited in New York, where the obesity prevalence is lower than that of many other regions in the United States. In future studies, the use of clinically relevant cutoffs for HOMA-IR could enhance the clinical applicability of research in this field. As discussed previously, due to the lack of available antibodies, IR isoform expression could not be assessed.

Overall, our results show that hyperinsulinemia is associated with IR expression in TNBC, and we hypothesize that this may be important for the development and progression of early stages of TNBC. The higher prevalence of hyperinsulinemia in Black women may be contributing to the higher observed rates of TNBC. However, further work is needed to determine if hyperinsulinemia increases IR expression in breast tissues, if IR activation is causative in the development of TNBC, the importance of the IR isoforms, the significance of nuclear IR expression in TNBC, and if strategies to reduce systemic hyperinsulinemia (such as sodium-glucose cotransporter 2 inhibitors or glucagon-like peptide 1 receptor agonists) will reduce the risk of developing TNBC.

## Data Availability

The data in this study are not publicly available, and restrictions apply to preserve patient confidentiality. The corresponding author will on request provide access to deidentified data upon approval of the Icahn School of Medicine at Mount Sinai Institutional Review Board.
